# Construction and analysis of the protein-protein interaction networks for schizophrenia, bipolar disorder, and major depression

**DOI:** 10.1186/1471-2105-12-S13-S20

**Published:** 2011-11-30

**Authors:** Sheng-An Lee, Theresa Tsun-Hui Tsao, Ko-Chun Yang, Han Lin, Yu-Lun Kuo, Chien-Hsiang Hsu, Wen-Kuei Lee, Kuo-Chuan Huang, Cheng-Yan Kao

**Affiliations:** 1 Department of Information Management, Kainan University, Taoyuan, Taiwan; 2 Department of Computer Science and Information Engineering, National Taiwan University, Taipei, Taiwan; 3 Graduate Institute of Biomedical Electronics and Bioinformatics, National Taiwan University, Taipei, Taiwan; 4 Graduate Institute of Electronics Engineering, National Taiwan University, Taipei, Taiwan; 5 Department of Psychiatry, Armed Forces Beitou Hospital, Taipei, Taiwan

## Abstract

**Background:**

Schizophrenia, bipolar disorder, and major depression are devastating mental diseases, each with distinctive yet overlapping epidemiologic characteristics. Microarray and proteomics data have revealed genes which expressed abnormally in patients. Several single nucleotide polymorphisms (SNPs) and mutations are associated with one or more of the three diseases. Nevertheless, there are few studies on the interactions among the disease-associated genes and proteins.

**Results:**

This study, for the first time, incorporated microarray and protein-protein interaction (PPI) databases to construct the PPI network of abnormally expressed genes in postmortem brain samples of schizophrenia, bipolar disorder, and major depression patients. The samples were collected from Brodmann area (BA) 10 of the prefrontal cortex. Abnormally expressed disease genes were selected by *t*-tests comparing the disease and control samples. These genes were involved in housekeeping functions (e.g. translation, transcription, energy conversion, and metabolism), in brain specific functions (e.g. signal transduction, neuron cell differentiation, and cytoskeleton), or in stress responses (e.g. heat shocks and biotic stress).

The diseases were interconnected through several “switchboard”-like nodes in the PPI network or shared abnormally expressed genes. A “core” functional module which consisted of a tightly knitted sub-network of clique-5 and -4s was also observed. These cliques were formed by 12 genes highly expressed in both disease and control samples.

**Conclusions:**

Several previously unidentified disease marker genes and drug targets, such as SBNO2 (schizophrenia), SEC24C (bipolar disorder), and SRRT (major depression), were identified based on statistical and topological analyses of the PPI network. The shared or interconnecting marker genes may explain the shared symptoms of the studied diseases. Furthermore, the “switchboard” genes, such as APP, UBC, and YWHAZ, are proposed as potential targets for developing new treatments due to their functional and topological significance.

## Background

Schizophrenia, bipolar disorder, and major depression are suffered by approximately 1%, 5% or 20%, respectively, of human during their life time. Improving the diagnoses and treatments of these devastating diseases is an important task. However, few studies of mental diseases have used post-mortem brain samples [1]. Researchers did not have convenient access to brain samples of psychiatric patients until 1994 when the Stanley Brain Collection started.

These three diseases each have distinct characteristics, but they also share a few symptoms. All diseases above may show signs of psychosis, and in which both bipolar disorder and major depression have depressive symptoms. The shared symptoms suggest related disease mechanisms. These diseases have always been affiliated with neuron and dopamine abnormalities [2-9]. Abnormalities in the glia, GABA, and other neurotransmitter systems were revealed in more recent studies using patients’ brain samples from the Stanley Brain Collections [10-15]. The genetics of these diseases are overlapped [16,17]. Related single nucleotide polymorphisms (SNPs) and mutations, such as coding variants in the lipid transporter ABCA13, are often associated with more than one of the three diseases [1,2]. Microarrays of frontal, prefrontal, cingulate, and cerebellar cortex samples show disruption of mRNA or protein expression in intracellular signalling, synaptic neurotransmission, oligodendrocyte, stress responses, cytoskeleton, ATP biosynthesis, and translation [18-35].

The data of human protein-protein interactions (PPIs) brought insights to the network biology of diseases and explained the interrelationships among disease-related genes and proteins. Recently, the schizophrenia markers, NRG1 and CACNG2, which were considered functionally un-related, were found to be connected *via* the ERGG and DRL protein families in PPI network [36]. Furthermore, in our pilot study, a potential schizophrenia marker, EXOC4, was identified by analyzing the PPI network constructed using four published schizophrenic marker genes [37].

This study constructed PPI networks for schizophrenia, bipolar disorder, and major depression using abnormally expressed genes in Brodmann area (BA) 10 of prefrontal cortex. The “core” functional module of BA10 was also constructed by the most highly expressed genes in disease and control samples. Potential disease marker genes and drug targets were also identified.

## Methods

This study constructed PPI networks for post-mortem prefrontal cortex of schizophrenia, bipolar disorder, and major depression patients. It focuses only on direct (physical) interactions among proteins. Genetic interactions were not investigated. The PPI networks were constructed based on the hypotheses that (1) the abundance of proteins and mRNAs were positively correlated in brains; (2) proteins were more likely to interact with proteins which had similar expression patterns or were more abundant; and (3) more abundant proteins participated in more active biological processes.

**Figure 1:**
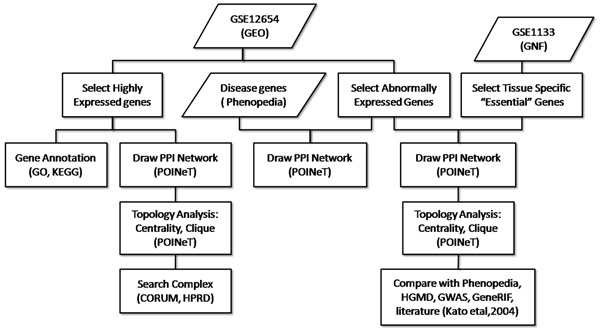
Research methodology

The research methodology is summarized in Figure 1. Microarray data series was used to identify genes abnormally expressed in patients’ BA10 of prefrontal cortex. These genes, together with the brain specific genes, were used to construct a PPI network for topological analyses. This network was compared with the PPI networks constructed by disease genes mentioned by in published literatures. The most abundant protein interactions in BA10 were revealed by the most highly expressed genes in the brain samples to outline the framework of prefrontal cortex biochemistry.

### Sources of microarray data

The raw data (CEL files) of microarray data series, GSE12654, were downloaded from Gene Expression Omnibus (GEO) and normalized by mas5 [38]. GSE12654 was first published by Iwamoto *et al* of RIKEN, Japan [39]. GSE12654 is the microarray data of post-mortem human brain sampled from the BA10 of four groups of people, including 13 schizophrenia patients, 11 bipolar disorder patients, 11 major depression patients, and 14 healthy controls [23]. Each group of the three diseases and the control samples was termed as a “sample group” in this study.

### Selection of the most highly expressed genes

The z-scores of genes were calculated within each disease or control sample group. Each gene had four z-scores—three for each disease sample groups and one for the control sample group. The genes with z-scores ≥ 1.96 in ≥ 49% samples of a given sample group (e.g. in ≥ 7/13 schizophrenia samples) were defined as the most highly expressed genes of the sample types. These genes were likely to encode the most abundant proteins in BA10 of patients or healthy people.

### Selection of tissue-specific “essential” genes for the healthy BA10 samples

GSE1133 (Human U133A/GNF1H) [40] was downloaded from the Novartis Research Foundation Gene Expression Database (GNF). The gcrma-normalized expression value of a gene in the prefrontal cortex was compared with the mean expression value of the same gene in all tissues examined in GSE1133. The genes were defined as prefrontal cortex-specific genes, if their expression values in the prefrontal cortex were > 4 fold higher than the mean values in all tissues. The genes which were both specific to the prefrontal cortex, as well as highly expressed (z-score ≥ 1.96 in ≥ 49% samples) in the control sample group of GSE12654, were defined as the tissue-specific “essential” genes of healthy BA10.

### Selection of abnormally expressed genes in disease samples

Considering the diverse conditions of post-mortem brain samples (e.g. pH values), the profiles of subjects (e.g. age, gender, and use of medication), and the complexity of disease mechanisms, the microarrays in GSE12654 were analyzed by 2-tailed *t*-test. Each disease sample group was paired with the control sample group in the *t*-tests. The genes which expressed abnormally in disease samples would be detected by the corresponding probes with significant changes (P values < 0.01) of signal intensities in the disease sample groups as comparing to the control.

### Construction of PPI networks for mental diseases

The most highly expressed genes and abnormally expressed genes were located on the human PPI network by an updated version of POINeT [37] to construct a query-query PPI (QQPPI) or level 1 PPI (L1PPI) networks. The updated version of POINeT contains the databases listed in Table 1 and has not been published. QQPPI networks only used the query marker genes as the nodes and revealed direct interactions among these queries. L1PPIs also showed the other non-query nodes directly connecting to the queries. L1PPI network allowed analysis of an extended network and could reveal indirect interactions (*via* common level 1 interactors as mediators) among query genes.

**Table 1:** PPI databases used for constructing PPI networks in this study

Database	Version or downloading date	Number of recorded PPIs
HPRD	Release 9	39194
BioGRID	3.1.70	356818
IntACT	2010-12-06	146227
NCBI gene interactions	2010-12-06	585982

### Topology analysis of PPI networks

To analyse the QQPPI and L1PPI networks, cliques and centralities were calculated. A clique is a set of genes (nodes) in which every two genes (nodes) are connected by a protein interaction (edge). Cliques have been used successfully to identify protein functional units in PPI networks [41]. Nodes within cliques are more likely to form complexes [42]. In this study, cliques with 4 nodes (clique-4) or above were identified from PPI networks. They were searched against CORUM [43] and HPRD [44] for potential protein complexes.

Centrality analyses can assist in identifying significant proteins (nodes) which have relatively more PPIs (edges) in a network. The centrality of nodes in the L1PPI network which consisted of the abnormally expressed and “essential” genes was calculated by CentiBiN [45,46]. The centrality scores were ranked. The ranks from different centrality algorithms were fused by summing the ranking numbers [47].

### Gene annotation

The highly expressed genes were classified into groups according to the Gene Ontology (GO) terms [48], using the Functional Classification tool in DAVID 6.7 [49]. Suitable parameters for classification stringency were chosen for the Gene Functional Classification tool as followed - a Kappa similarity term overlap of 3, a Kappa similar threshold of 0.3, an initial group membership of 3, a final group membership of 3, and a multiple linkage threshold of 0.5.

The functions of abnormally expressed disease genes (P value < 0.01) were summarized using the FatiGO+ module of Babelomics 4.2 [50,51]. Functional enrichments were performed for terms in REACTOME, GO cellular localisation, GO biological process, or GO molecular function with default parameters. The functions of genes were annotated by the Gene List Report in DAVID 6.7 [49].

### Comparison with the study of Iwamoto [39]

Different analytical methods of microarray data often reveal similar but not identical results. The original contributors of microarray GSE12654 were Iwamoto *et al* who focused on gender- and age-related genes in their analysis. The disease genes identified from GSE12654 by Iwamoto *et al*[39] were constructed into QQPPI and L1PPI networks and compared with ours.

### Comparison with data from Phenopedia, GWAS, HGMD, and GeneRIF

Microarray studies identify changes in gene expression patterns, whereas gene association studies focus on the identification of disease-related SNPs and mutations. The disease markers revealed by these different approaches were compared with our study.

Phenopedia collect genes which associate with human diseases by retrieving curated records from PubMed on a weekly basis since 2001 [52]. The genes which were listed in Phenopedia as being associated with schizophrenia, bipolar disorder, and major depression were constructed into QQPPI and L1PPI networks, and compared with the network constructed by the abnormally expressed disease genes. The disease-associated SNPs and mutations which were listed in A Catalog of Published Genome-Wide Association Studies (GWAS) [53] or The Human Genome Mutation Database (HGMD) [54] were also compared with our findings.

In addition, dopamine, GABA, and glutamate are three of the most significant chemicals known to affect the symptoms of the studied mental diseases. The association of abnormally expressed and tissue-specific “essential” genes with dopamine, GABA, and glutamate were searched in GeneRIF.

## Results

BA10 of the prefrontal cortex is believed to be responsible for cognition, which is a function disrupted often in patients of psychiatric diseases [55,56]. By analyzing the microarrays of human BA10 samples, this study identified potential disease marker genes and drug targets of schizophrenia, bipolar disorder, and major depression. Additionally, a “core” functional module was constructed using the genes which were not only highly expressed in both disease and control samples, but also topologically significant in the PPI networks.

### Abnormal gene expression in the prefrontal cortex of schizophrenia, bipolar disorder, and major depression patients

**Figure 2:**
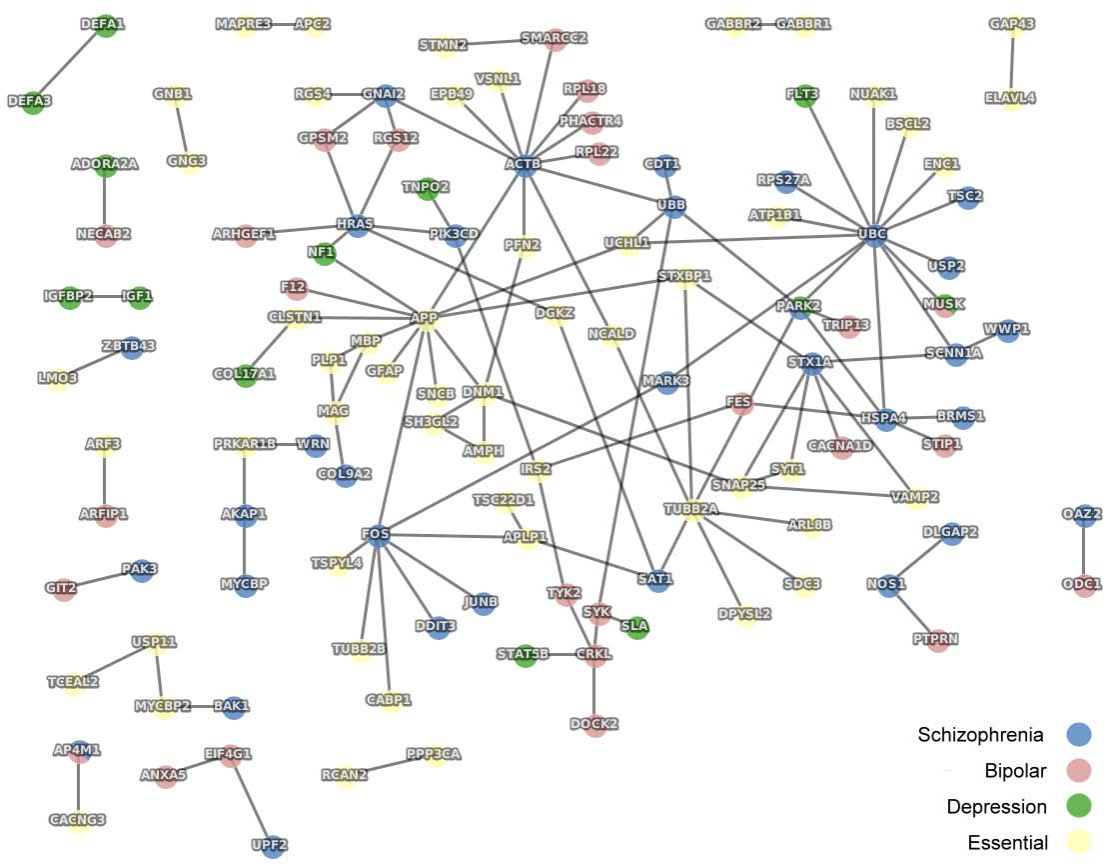
QQPPI constructed by abnormally expressed disease genes and tissue-specific “essential” genes

The differential expression of genes in the BA10 samples was investigated by *t*-tests comparing the signal intensities of corresponding probes in microarrays for the disease and control samples. The genes of which the corresponding probes had P values < 0.01 were defined as abnormally expressed and proposed as disease markers. These disease markers, together with the tissue-specific “essential” genes, were used as queries to reveal the QQPPI network shown in Figure 2. The functions of query genes are listed in Additional file 1 (titled „Abnormally expressed disease genes and functions'). The genes and P values of corresponding probes are listed in Additional file 2, 3, and 4 (titled „Abnormally expressed genes and corresponding probe IDs and P values in *t*-tests’ for „schizophrenia’, „bipolar disorder’, and „major depression’, respectively).

The abnormally expressed genes could be classified into 13 groups according to their functional annotations as detailed in Additional file 5 (titled „GO functional classification for abnormally expressed genes'). They participated in transcription, translation, cytoskeleton, neuron differentiation, ATP-binding, cellular transportation, and many other biological processes. Using the functional enrichment tool FATIGO+, a few functional terms were found to be more abundant in our abnormally expressed proteins of BA10 than in the entire proteome encoded by human genome. Table 2 lists the GO biological processes which were enriched in our abnormally expressed genes. The results of functional enrichment analyses for GO cellular compartment, GO molecular function, and REACTOME are detailed in Additional file 6 (titled „FatiGO term enrichment with P values smaller than 0.01). Enriched functions can be classified into five groups—(1) neuron and signal transduction-related, such as neuron projection, transmission of nerve impulse, synaptic transmission, synaptogenesis, and signalling by NGF; (2) cytoskeleton; (3) gene expression, such as translation and ribosomes; (4) metabolisms of lipids, lipoproteins, proteins, polyamines, and sugars (diabetes); and (5) stresses, such as influenza infection. The enrichment of neuron and signal transduction-related functions was expected. Abnormality in translation-related genes has been observed in previous studies [39,57]. Abnormality in cytoskeleton genes could lead to disrupted cellular mobility of Golgi apparatus, which has a place in neuron signal transduction [58-61]. Previous studies have also shown abnormal expression of ATP-related or mitochondrial genes [20,22,62-64]. The enriched metabolism functions can be further evidences of abnormal energy conversion in patients' prefrontal cortex. The relationship to influenza infection can be explained as biotic stress; and stresses have often been reported as inducers of mental diseases [65-67].

**Table 2:** Functional enrichment analysis for abnormally expressed disease markers

GO: biological process	Go term ID	Genes with this term
Translation	GO:0006412	DHX29, EIF4G1, FAU, GSPT1, KRT7, MAZ, RPL10A, RPL17, RPL 18, RPL21, RPL21P119, RPL21P16, RPL21P39, RPL21P80, RPL21P93, RPL21P97, RPL22, RPS15A, RPS5, VARS, MRPS12, PABPC4, RPS27A, TOP3A

Transmission of nerve impulse	GO:0019226	CPNE6, GABARAP, MUSK, POU3F2, VGF, ADORA2A, GRM8, NF1, NMU, PARK2, DLGAP2, GNAI2, HRAS, NOS1, RPS27A, STX1A, UBB, UBC

Synaptic transmission	GO:0007268	CPNE6, GABARAP, MUSK, VGF, ADORA2A, GRM8, NMU, PARK2, DLGAP2, GNAI2, HRAS, NOS1, RPS27A, STX1A, UBB, UBC

Negative regulation of catalytic activity	GO:0043086	GNAT1, TNNI3, ADORA2A, GRM8, NF1, GNAI2, OXA1L, RPS27A, SH3BP5, TSC2, UBB, UBC

Regulation of synaptic transmission	GO:0050804	VGF, ADORA2A, GRM8, NMU, PARK2, GNAI2, HRAS, RPS27A, STX1A, UBB, UBC

Translational elongation	GO:0006414	FAU, RPL10A, RPL17, RPL18, RPL21, RPL21P119, RPL21P16, RPL21P39, RPL21P80, RPL21P93, RPL21P97, RPL22, RPS15A, RPS5, VARS, RPS27A

Regulation of synaptogenesis	GO:0051963	MUSK, RPS27A, UBB, UBC

Polyamine metabolic process	GO:0006595	ODC1, SLC6A11, OAZ2, SAT1

**Figure 3:**
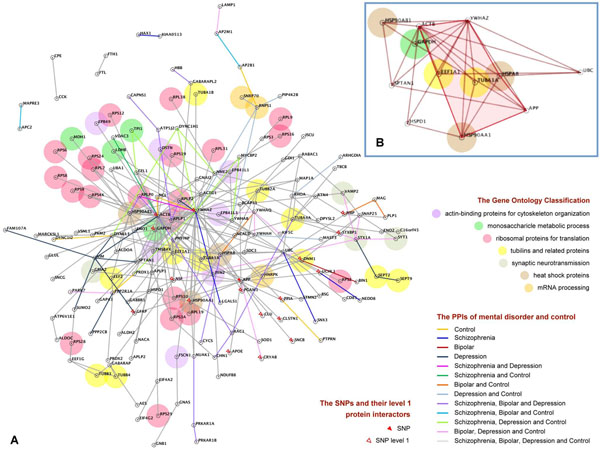
a) QQPPI constructed by highly expressed genes in disease and control samples; b) The “core” functional module

The ranks of centrality were calculated by various algorithms and are listed in Additional file 7 (titled „Centrality analysis of abnormally expressed genes in QQPPI network'). The top ranked nodes are summarised in Table 3. Proteins which rank higher in centrality analyses of PPI networks usually have more crucial biological functions [68]. The top ranked nodes (e.g. UBC, UBB, and ACTB) were therefore proposed as having critical roles in disease mechanisms. Only three clique-3s were found (data not shown). Clique-4 or above was not identified as a result of the looser network formed by the abnormally expressed genes (Figure 2) in comparison with the tightly knitted network formed by the highly expressed genes (Figure 3).

**Table 3:** Centrality analysis of PPI network

Gene symbol	Disease(s) or essential gene*	Gene function(s)	Sum of ranks**
UBC	S	• ubiquitin C	7
ACTB	S	• actin, beta	15
UBB	S	• ubiquitin B	29
APP	E	• amyloid beta (A4) precursor protein	32
FOS	S	• v-fos FBJ murine osteosarcoma viral oncogene homolog	51
HSPA4	S	• heat shock 70kDa protein 4	54
PARK2	SD	• Parkinson disease (autosomal recessive, juvenile) 2, parkin	67
SYK	B	• spleen tyrosine kinase	97
TUBB2A	E	• tubulin, beta 2A	117
TSC2	S	• tuberous sclerosis 2	127
UCHL1	E	• ubiquitin carboxyl-terminal esterase L1 (ubiquitin thiolesterase)	217
MARK3	S	• MAP/microtubule affinity-regulating kinase 3	236
RPS27A	S	• ribosomal protein S27a pseudogene 12; ribosomal	355
DNM1	E	• dynamin 1	476
IRS2	E	• insulin receptor substrate 2	497
GNAI2	S	• guanine nucleotide binding protein (G protein), alpha inhibiting activity polypeptide 2	519
SMARCC2	B	• SWI/SNF related, matrix associated, actin dependent regulator of chromatin, subfamily c, member 2	525
HRAS	S	• v-Ha-ras Harvey rat sarcoma viral oncogene homolog	527
SAT1	S	• spermidine/spermine N1-acetyltransferase 1	529
CRKL	B	• v-crk sarcoma virus CT10 oncogene homolog (avian)-like	531

The L1PPI network (not shown) was constructed by abnormally expressed disease markers, tissue-specific “essential” genes

*The listed genes were found abnormally expressed in samples of schizophrenia (S), bipolar disorder (B) or major depression (D), or were tissue-specific “essential” (E) genes.

**The sum of centrality ranks calculated using various centrality algorithms for the disease and “essential” genes shown in Figure 2.

### The disease marker genes identified through different analytical approaches were connected in PPI networks

The microarray data series used in this study has been analysed by the original contributors, Iwamoto *et al*[39]. In the studied mental diseases, Iwamoto *et al* revealed down-regulation of receptor-, transporter-, and channel-encoding genes; and up-regulation of transcription-, translation-, stress- and molecular chaperon-related genes [39]. Although the genes identified in our studies were not identical to the findings of Iwamoto *et al*, they fall into similar functional categories. Although few of our disease markers had direct protein interactions with the ones described in Iwamoto *et al*[39], many had indirect interactions through mediator proteins (level 1 interactors) in between. Taking bipolar disorder as an example, there was only one gene listed in both Iwamoto *et al* and this study as a disease marker; only two of our abnormally expressed genes for bipolar dirorder had direct interactions (PPIs) with two bipolar disorder markers of Iwamoto *et al*. However, our markers of bipolar disorder shared 120 level 1 interactors with the bipolar disorder markers of Iwamoto *et al*[39].

Similar results were observed in the data of Phenopedia as summarized in Table 4. For schizophrenia, bipolar disorder, and major depression, there were only 11, 5, and 5 genes, respectively, that were listed both as disease genes in Phenopedia and as our disease markers. Interestingly, there were many PPIs formed between the Phenopedia disease genes and our abnormally expressed genes for schizophrenia, bipolar disorder, and major depression as illustrated in Additional file 8, 9, and 10 (titled „Interrelationship between Phenopedia and abnormally expressed genes' in „schizophrenia', „bipolar disorder', and „major depression', respectively). The names of these disease genes are listed in Additional file 11 (titled „Comparison of disease genes listed in Phenopedia, HGMD, and GWAS'). The diseases associated SNPs and mutations which are listed in GWAS and HGMD were also compared with the abnormally expressed genes identified in this study and listed also in Additional file 8.

**Table 4:** Comparison to the disease genes listed in Phenopedia

Record type	Schizophrenia	Bipolar disorder	Major depression
Disease genes in Phenopedia (P)	1001	722	226
Abnormally expressed genes (T)*	110	100	44
Both P and T (shared nodes)	11	5	1
PPIs between P and T	252	73	11

* Genes with P values < 0.01 in *t*-tests

Furthermore, dopamine, GABA, and glutamate are critical chemicals affecting the symptoms of schizophrenia, bipolar disorder, and major depression. Genes which were described in GeneRIF as involving in the biochemistry of these chemicals are listed in Table 5. This data was considered as the indirect evidence to the association of genes to the studied mental diseases.

**Table 5:** Genes which participated in the biochemistry of GABA, glutamate, or dopamine

Gene Symbol	GABA	Glutamate	Dopamine
APP	**+**	**+**	**+**
GABBR2	**+**	**+**	
GABBR1	**+**	**+**	
SLC12A5	**+**		
GABARAP	**+**		
SLC1A3		**+**	
NOS1		**+**	
GRM8		**+**	
GLUL		**+**	
UCHL1			**+**
SYNGR3			**+**
STXBP1			**+**
STX1A			**+**
SNCB			**+**
PRKAR1B			**+**
PARK2			**+**
IGF1			**+**
GPR143			**+**
EPB41L1			**+**
CRYAA			**+**
ADORA2A			**+**

### The highly expressed genes and the most abundant protein interactions in the prefrontal cortex

In order to identify the most abundant protein interactions in BA10, the PPI network of highly expressed genes were constructed for schizophrenia, bipolar disorder, major depression, and healthy control samples as illustrated in Figure 3a. Many PPIs were shared by two or more of the diseases. Disease-associated SNPs, mutations, and GO functional classes were identified for each gene and labelled as shown in Figure 3a.

In this PPI network, there were one clique-5 and several clique-4s that tightly knitted into a sub-network containing 12 genes as shown in Figure 3b. These 12 genes in the BA10 cliques encode four heat shock proteins (HSPA8, HSP90AA1, HSPD1, HSP90AB1), the tubulin protein (TUBA1A), the ubiquitin protein (UBC), the actin-binding cytoskeleton protein (SPTAN1), beta-actin (ACTB), the energy generating glyceraldehyde-3-phosphate dehydrogenase (GAPDH), translation elongation factor (EEF1A1), signal transduction protein (YWHAZ), and amyloid beta (A4) precursor protein (APP). This sub-network of 12 highly expressed and topologically significant genes was proposed as the “core” functional module of BA10 for both patients and healthy people.

## Discussion

This study combined data of quantitative transcriptomics and PPIs for statistical and topological analyses. New disease marker genes and potential drug targets were revealed. Shared disease mechanisms, although hypothetical at the present, were proposed based on common marker genes and interconnecting PPIs to explain the shared symptoms among diseases. A “core” functional module of BA10 was also proposed.

### Disease markers and potential drug targets

The genes which had P values < 0.01 in our *t*-tests were defined as abnormally expressed in disease samples. These genes were proposed as disease marker genes and constructed into a PPI network as illustrated in Figure 2.

Previous studies have shown that the probes (and the genes which they represented) with lower P values appeared as more effective clustering features for separating the disease samples from the controls in hierarchical clusters (unpublished data). The observation suggested that genes with lower P values are more important disease markers.

The genes with the lowest P values in our *t*-tests were not well-studied genes. SBNO2 was a gene of which the probe had the lowest P value in the *t*-test comparing schizophrenia and control samples. SBNO2 has a strawberry notch homolog in fruit fly. The gene is involved in the anti-inflammatory signalling pathway [69]. It has also been associated with type 2 diabetes mellitus, of which shared many disease genes with mental diseases [52,70]. SEC24C was a gene of which the probe had the lowest P value in the *t*-test comparing bipolar disorder and control samples. SEC24C encodes a protein which may be involved in ER to Golgi vesicular transportation [71]. It has been associated another mental disease, Alzheimer's disease [52]. SRRT was a gene of which the probe had the lowest P value in the *t*-test comparing major depression and control samples. SRRT is possibly involved in transcriptional regulation and RNA metabolism as it is a homolog to an *Arabidopsis* serrate RNA effector [72]. Apart from this study, SBNO2, SEC24C and SRRT have never been associated with schizophrenia, bipolar disorder, or major depression; these genes did not form PPI with any of our abnormally expressed marker genes, either.

There appeared a weak negative correlation between the P values of a gene (probe) and its centrality ranks in this study. More “essential” proteins in PPI networks have also been shown to rank higher than less important proteins in centrality analyses [68,73]. Interestingly, the nodes which ranked highest in the centrality studies were mostly schizophrenia markers as listed in Table 3. The top ranked genes in centrality analysis, UBC, ACTB, and UBB, were all abnormally expressed in schizophrenia samples. UBC and UBB encode the polyubiquitin precursors. ACTB encodes the beta-actin protein. The roles of these proteins in mental disease mechanisms are not clear. None of these three genes have been associated with any mental disease; but UBC has been associated also with type 2 diabetes mellitus [70].

Schizophrenia samples had the largest number of abnormally expressed genes, and major depression had the fewest. The above observations aligned with the most severe and complex symptoms of schizophrenia in comparison to the other two diseases; major depression encompassed the least of symptoms. All three disorders may show signs of psychosis, but symptoms such as hallucinations and poverty of speech are usually only seen with schizophrenia. The delusions associated with bipolar disorder and major depression is usually the milder “mood-congruent” delusion contributing to the overly positive or negative self perceptions of manic or depressive patients. Whereas the delusions occur with schizophrenia can be completely implausible and “bizarre”. The additional symptoms of schizophrenia may be caused by additional aberrations of genes. It must be noted, however, that few evidences are available to support or clarify this assumption.

### Shared disease mechanisms

The three studied mental diseases are genetically related and share disease genes [16,17]. Each disease may have malfunctions in multiple biological pathways, and the same malfunctions may occur in multiple diseases [74]. There are also increasing numbers of evidences which indicate correlations in genetic risks for schizophrenia and bipolar disorder [75-79].

A few genes were found to be abnormally expressed in more than one disease in this study. As shown in Figure 2, MUSK, which encodes a protein responsible for the assembly of receptors in post-synaptic neuromuscular junctions, was abnormally expressed in samples of bipolar disorder and major depression [80,81]. PARK2, a gene of unknown function associated with related phenotypes such as Parkinson disease, nerve degeneration, cognition disorders, visual perception, attention, and memory, was abnormally expressed in schizophrenia and major depression [52,82-84]. AP4M1, which encodes a subunit of AP-4 complex responsible for transportation of proteins from Golgi, was abnormally expressed in schizophrenia and bipolar disorder [52,85]. Many of the genes were also found to be highly expressed in more than one disease as shown in Figure 3a.

One might hypothesize that the shared genes are the reasons for shared symptoms— the abnormal expression of MUSK may cause the depression of bipolar disorder and major depression; PARK2 may cause the psychosis symptoms such as delusions, avolition, blunted effect, asociality, and cognitive dysfunction, which are commonly seen with varying severities in both schizophrenia and major depression; AP4M1 may cause the psychosis symptoms such as the less “bizarre” forms of delusions and restlessness sometimes observed in both schizophrenia and bipolar disorder. These hypotheses are far from being conclusive. Further researches such as mutant studies in animal models, genetic association studies, and gene and protein expression analyses are required to better explain the observed phenomenon of overlapping PPI network of the mental diseases. Unfortunately, as symptoms such as hallucinations and delusions are uniquely human, the exact roles of disease-associated genes play in mental disease mechanisms are difficult to be studied using mutant model animals [86].

The centrality ranks of MUSK, PARK2, and AP4M1 might indicate the divergence or similarity of the studied mental diseases, if phenotypical similarities were positively correlated with similarities in disease mechanisms. In the PPI network constructed using 3550 human disease genes retrieved from OMIM Morbid Map, shared genes were more central than disease-specific genes, and the genes shared by phenotypically similar diseases are less central than the ones shared by phenotypically divergent diseases [73]. AP4M1 ranked the lowest in centrality among the three genes as shown in Fig. 2; PARK2 ranked the highest. We might deduce that the disease mechanisms of schizophrenia and bipolar disorder are most similar among the three studied diseases; and the disease mechanisms of schizophrenia and major depression are most diverged.

### “Switchboards” in PPI sub-networks of psychiatric diseases

Apart from shared marker genes, marker genes of multiple diseases sometimes interact with the same critical nodes. These critical nodes were designated as “switchboard” nodes to describe their place in interconnecting PPIs of different diseases. The “switchboards” usually ranked higher in centrality analyses, suggesting they are more “essential” and effect diverged ranges of phenotypes. The abnormalities of different protein interactions with the same gene may explain the relevant but different symptoms of the studied diseases.

In the schizophrenia PPI network shown in figure 2, the “switchboard” APP interacted with the abnormally expressed ACTB and FOS. APP was a tissue-specific “essential” gene, which encodes a highly expressed beta-amyloid precursor protein. ACTB encodes the beta-actin, which is responsible for cellular structure and (neuron signal) mobility [87-89]. FOS is likely to associate with cell differentiation, apoptotic cell death, and depression-related diseases such as bulimia and anorexia [52,90]. In bipolar disorder, APP interacted with the abnormally expressed F12. F12 encodes a coagulation factor which circulates in blood as zymogen. Mutations in F12 may prolong whole-blood clotting time; and the gene has been associated with type 1 and 2 diabetes mellitus [52,91-93]. In major depression, APP interacted with the abnormally expressed NF1. NF1 encodes a neurofibromin responsible for signal transduction, and has been associated with mental retardation and autism [52,94,95]. APP has been listed in the HGMD as being associated with schizophrenia [96]. However, the transcript of APP was consistently highly abundant in all disease and control samples. The mutation of APP in patients might not have affected its transcription.

A “switchboard” can also be a disease gene. For example, the ubiquitous protein, UBC, was abnormally expressed in schizophrenia samples and interacted with the maker genes of schizophrenia (i.e. TSC2, SCNN1A and USP2), bipolar disorder (i.e. MUSK), and major depression (i.e. MUSK and FLT3).

The same “switchboard” mechanism was observed in the network constructed by the most highly expressed genes. One such example was YWHAZ, which encodes a signal transduction protein. YWHAZ interacted with 43 nodes as shown in Figure 3a and was highly expressed in disease and control samples. The abnormal PPIs (i.e. PPIs of highly expressed genes that did not occur in the controls) between YWHAZ and many other proteins were observed in disease samples. The interaction with NCL was abnormal in schizophrenia, bipolar disorder, and major depression. The other YWHAZ abnormalities were the interactions with RNPS1 and LGALS1 in schizophrenia; the interactions with MYCBP2, PRDX1 or TP1l in bipolar disorder; the interactions with RPLP2 and VIM in major depression; and the interactions with RPLP0 in both schizophrenia and major depression.

### The “core” functional module

We proposed that the 12 genes in the clique-5 and -4s in PPI constructed by the highly expressed genes were central to the functioning of BA10 (Figure 3a and b). These nodes were the ones with the highest ranks in the centrality analysis of PPI network of highly expressed gene. A few genes, such as UBC and ACTB, were also highly ranked in the centrality analysis of the abnormally expressed PPI network as listed in Table 3. Many of these genes encode members of important protein complexes as summarised in Table 6. They were mostly tissue-specific “essential” genes and highly expressed in all three studied mental diseases as well as healthy control. The nodes in cliques were sometimes involved in biological processes which were disrupted in schizophrenia, bipolar disorder, or major depression.

**Table 6:** Cliques in the PPI network constructed by highly expressed genes in disease and control samples

List of clique-4				Protein complex
HSPA8	HSP90AA1	APP	YWHAZ	**•** Nil
HSPA8	HSP90AA1	APP	ACTB	**•** Amyloid beta protein oligomer (PMID:15834427)
HSPA8	HSP90AA1	ACTB	YWHAZ	**•** APP-FE65-LRP complex (PMID:15115822)
HSPA8	APP	ACTB	YWHAZ	**•** APP-TIMM23 complex (PMID:16943564)
HSP90AA1	APP	ACTB	YWHAZ	**•** APP-TOMM40 complex (PMID:16943564)
HSP90AA1	ACTB	YWHAZ	TUBA1A	**•** Emerin architectural complex (PMID:17620012)
GAPDH	HSP90AB1*	ACTB	YWHAZ	**•** Emerin complex 25 (PMID:17620012)
GAPDH	APP	ACTB	YWHAZ	**•** Emerin-actin-NMI- (PMID:alphaII) spectrin complex (PMID:17620012)
HSPA8	ACTB	UBC*	YWHAZ	**•** Kinase maturation complex 1 (PMID:14743216)
HSP90AB1*	ACTB	SPTAN1	YWHAZ	**•** P2X7 receptor signaling complex (PMID:11707406)
HSP90AA1	HSPD1	ACTB	YWHAZ	**•** Profilin 2 complex (PMID:9463375)
EEF1A1*	ACTB	UBC*	YWHAZ	**•** Profilin 2 complex (PMID:9463375)
APP	ACTB	SPTAN1	YWHAZ	**•** Nil

The protein complexes which may contain nodes within these cliques-4s are listed.

* This protein was not documented as a member of the suggested protein complex.

### Teams of disease marker genes

The abnormally expressed genes identified in this study were compared with published disease associated genes from previous analysis of the same data series (GSE12654), Phenopedia, GWAS, and HGMD. Although few genes were consistently identified in diseases by different groups of researchers, many of these genes were found to form QQPPIs or share the same level 1 PPI interactors as shown in Table 4 and Additional file 8, 9, and 10.

The observations above suggested that, in brains of patients, disease genes can become defected due to various abnormalities and lead to the same symptoms. Each research approach may only detect a sub-set of abnormalities, such as mutation, significant changes in gene expression, or smaller changes in gene expression. Besides, only a snapshot of transcript abundance can be detected in post-mortem brain samples. The proteins were likely to work in teams—the failure of any team members at any given time may similarly disrupt the participating biological process and cause similar phenotypes. A protein team may be a set of proteins which have direct physical interactions, such as QQPPIs, or *via* a common protein, such as in the more extended L1PPIs. A more stringent definition of a protein team can be a set of proteins which form cliques. The teams may also be defined by genetic interactions, although it may not be applicable for human samples which cannot have synthetic lethal experiments; nonetheless, redundancy or overlapping of gene functions may be speculated with sequence homology.

### Network medicine

Psychiatric drugs may be developed based on the concepts of network medicine. Network analysis of disease genes have been shown to significantly accelerate the trials for new treatments [97].

Combining drugs to target the largest number of disease genes in a PPI network, while avoiding non-disease genes to avoid side effects, has been shown to create effective new treatments for complex diseases [98]. Besides, topologically significant non-markers can also be potential drug targets so that the “switchboard” gene APP and its neighbouring nodes were proposed as potential drug targets [99]. APP is a cell surface receptor and trans-membrane precursor which can be cleaved to from peptides. The exact function of APP was not clear, but its roles in cellular signalling neuronal adhesion and positioning in cortical layers have been observed in mice models [100,101]. APP has also been reported to participate in the biochemistry of GABA, dopamine, and glutamate (Table 5), which are all known to have significant places in the symptoms of the studied mental diseases. Abnormal accumulation of APP protein has long been associated with Alzheimer's disease [102]. The polymorphism of APP has also been associated with schizophrenia as listed in HGMD, although its gene expression was not significantly different in disease samples of this study. The gene's association with cognition, dementia, and type 2 diabetes mellitus have also been mentioned [52]. The other “switchboards”, including UBC and YWHAZ, and their neighbouring nodes can also be potential targets for developing new treatments.

### Limitations of the research methods

This study analysed the disease mechanisms by considering the interactions of the proteins and employing topological analyses. However, the extensiveness of PPI subnetworks was limited by the availability of PPI recorded in the PPI databases listed in Table 1. Markers whose PPI data were not recorded in databases would be excluded in the proposed PPI networks. The incomplete human PPI network could lead to incomplete PPI network for the disease samples; it could also bias the topological analyses. The significance of disease markers was therefore evaluated by both *t*-tests and centrality analyses. Besides, abnormalities such as deformed protein structures were not observable in this study. This study only proposed disease markers which can be detected by examining the transient abundances of mRNAs. The functions of many disease marker genes were speculated by homologous genes in other model organisms and required confirmation.

## Conclusions

By identifying abnormally expressed genes in post-mortem brain samples of mental disease patients, several disease marker genes were proposed for schizophrenia, bipolar disorder, and major depression. The disease markers were constructed into PPI networks and analysed by topological theories.

A few genes were shared among the studied diseases, such as MUSK, PARK2, and AP4M1. They are evidences of shared disease mechanisms. The studied diseases also shared disease genes with the other mental diseases, such as Alzheimer's and Parkinson, and metabolic diseases, such as type 1 and 2 diabetes mellitus. The research methodology of this study may be applied to expand our investigations to related diseases. Genes with higher P values, ranked lower in centrality analyses and not shared among diseases are proposed as more effective disease marker genes; the abnormal expression of these genes are more likely to be unique to a specific disease.

Nevertheless, disease markers which ranked higher in centrality analyses, interacted with the “switchboards”, or were members of the “core” functional module, were considered to have more “essential” roles in the disease mechanisms. These genes included SBNO2, CEACAM5, AKAP1, UBC, ACTB, UBB, and FOS for schizophrenia; SEC24C, PGLYRP1, ARHGAP4, RPL22, SLC6A11, and SYK for bipolar disorder; and SRRT, PARK2, LILRA4, STK17A, IGFBP2, and NF1 for major depression. These markers, together with the “switchboards” such as APP, UBC, and YWHAZ, were proposed as targets for drug development.

The three studied mental diseases showed aberrations in common biological processes. Most of the disease markers fall into the functional categories which have been previously proposed as being related to the three studied mental diseases. Based on published data and the results of this study, it might be said that the stress responses, cellular signal transduction, neuron cell differentiation and aging, energy metabolism, and translation were dysfunctional in patients suffering schizophrenia, bipolar disorder, or major depression. However, we are still far from drawing a clear picture of the molecular biology of patients’ brains. Extensive studies are still required for establishing the disease mechanisms.

## List of abbreviations used

BA: Brodmann Area; Bi: bipolar disorder; Co: control; De: major depression; GEO: Gene Expression Omnibus; GO: Gene Ontology; GOA: Gene Ontology Annotation; GWAS: A Catalog of Published Genome-Wide Association Studies; HGMD: The Human Genome Mutation Database; L1PPI: level 1 protein-protein interaction; LDL: low-density lipoprotein; PPI: protein-protein interaction; QQPPI: query-query protein-protein interaction; Sc: schizophrenia; single SNP: nucleotide polymorphism.

## Authors' contributions

SAL conceived the study, programmed the bioinformatics analysis tools, carried out the data analysis and assisted in drafting the manuscript. TTHT conceived the study, interpreted the results, drafted the manuscript, surveyed the microarray data, and contributed to the design of the bioinformatics analysis tools. KCY assisted in the interpretation of results, drafting of the manuscripts, and contributed to the design of the bioinformatics analysis tools. LH programmed the bioinformatics analysis tools and carried out the data analysis. YLK and CHH assisted in data analyses. WKL assisted in the interpretation of results. KCH contributed to drafting the manuscript, interpretation of the data, and project management. CYK conceived the study and participate in coordination and management of the research project.

## Conflict of interest

The authors confirm that they have no conflict of interest.

## Supplementary Material

Additional file 1 Abnormally expressed disease genes and functions Click here for file

Additional file 2 Abnormally expressed genes and corresponding probe IDs and P values in *t*-tests for schizophrenia Click here for file

Additional file 3 Abnormally expressed genes and corresponding probe IDs and P values in *t*-tests for bipolar disorder Click here for file

Additional file 4 Abnormally expressed genes and corresponding probe IDs and P values in *t*-tests for major depression Click here for file

Additional file 5 GO functional classification for abnormally expressed genes Click here for file

Additional file 6 FATIGO term enrichment with P values smaller than 0.01 Click here for file

Additional file 7 Centrality analysis of abnormally expressed genes in QQPPI network Click here for file

Additional file 8 Interrelationship between Phenopedia and abnormally expressed genes in schizophrenia Click here for file

Additional file 9 Interrelationship between Phenopedia and abnormally expressed genes in bipolar disorder Click here for file

Additional file 10 Interrelationship between Phenopedia and abnormally expressed genes in major depression Click here for file

Additional file 11 Comparison of disease genes listed in Phenopedia, HGMD, and GWAS Click here for file
