# Supplementary figures and images for: Construction and analysis of the protein-protein interaction networks for schizophrenia, bipolar disorder, and major depression
Source: BMC Bioinformatics. 2011 Nov 30;12(Suppl 13):S20. doi: 10.1186/1471-2105-12-S13-S20 (PMC3278837; doi:10.1186/1471-2105-12-S13-S20)

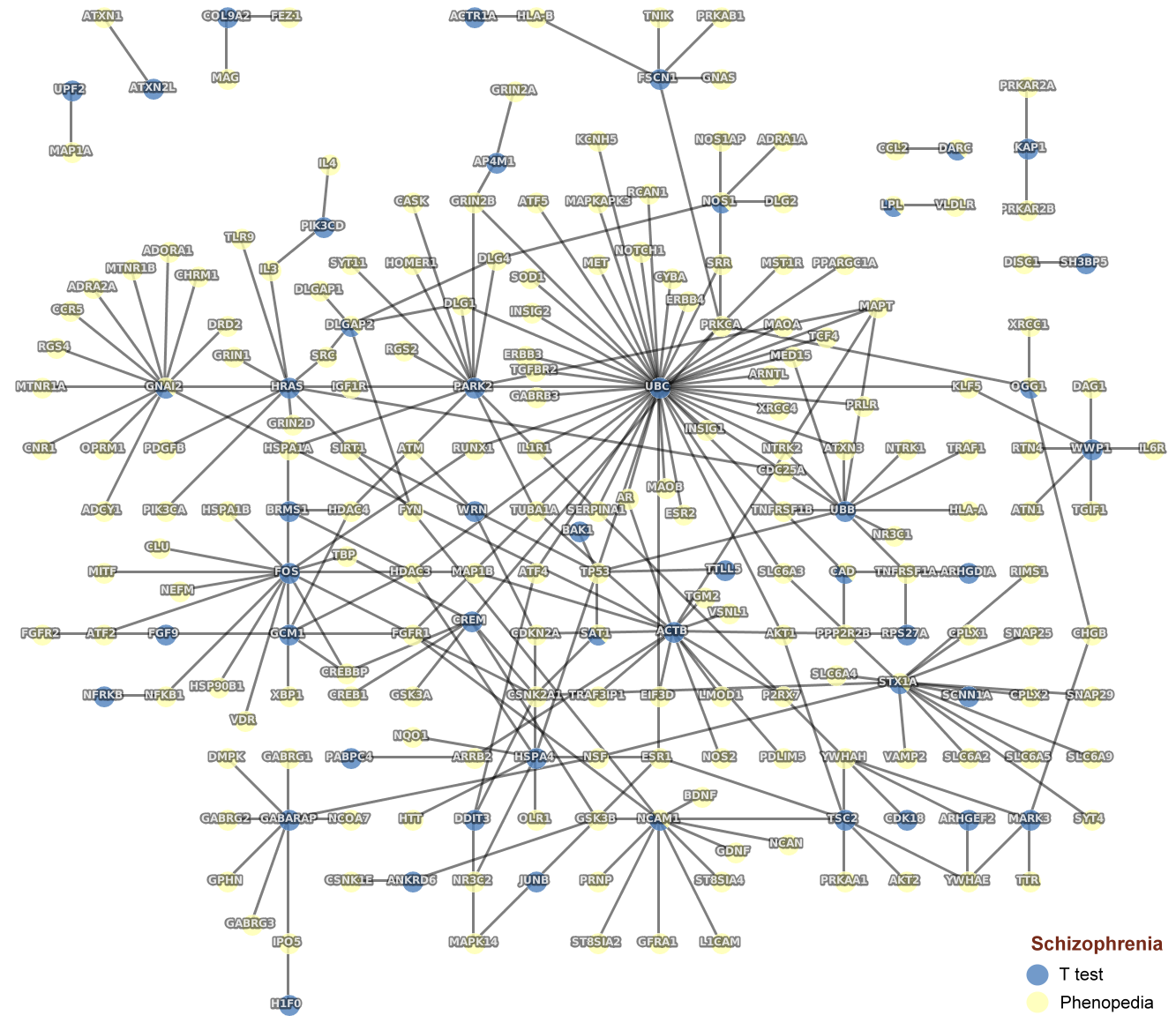

Supplement: Additional file 8 — Interrelationship between Phenopedia and abnormally expressed genes in schizophrenia [file 1471-2105-12-S13-S20-S8.png]

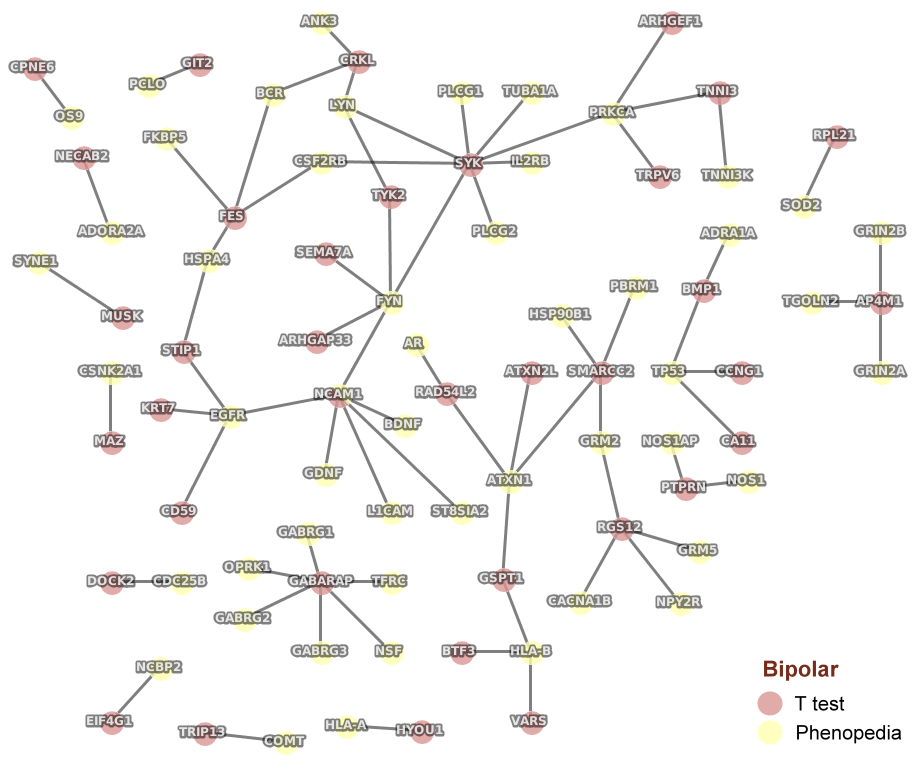

Supplement: Additional file 9 — Interrelationship between Phenopedia and abnormally expressed genes in bipolar disorder [file 1471-2105-12-S13-S20-S9.png]

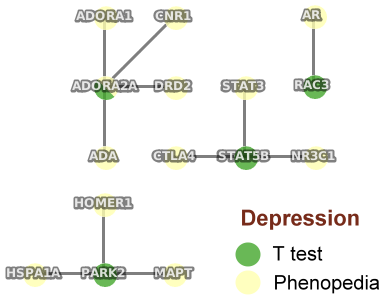

Supplement: Additional file 10 — Interrelationship between Phenopedia and abnormally expressed genes in major depression [file 1471-2105-12-S13-S20-S10.png]
